# Mode of birth after caesarean section: individual prediction scores using Scottish population data

**DOI:** 10.1186/s12884-019-2226-6

**Published:** 2019-02-28

**Authors:** Sara Helen Denham, Tracy Humphrey, Claire deLabrusse, Nadine Dougall

**Affiliations:** 1 School of Health and Social Care, Edinburgh Napier University, Sighthill Campus, Edinburgh, EH11 4BN UK; 2 School of Health Sciences (HESAV) Midwifery Department, University of Applied Sciences and Arts Western Switzerland, Lausanne, Switzerland

## Abstract

**Background:**

Rising caesarean section (CS) rates are a global health concern. Contemporary data indicates that almost 50% of CS are electively performed, with a high proportion of these being a repeat procedure. Vaginal birth after caesarean (VBAC) is recognised as a safe way to give birth in developed countries. UK national maternity policy and worldwide professional guidance supports shared decision-making about mode of birth with women following CS. Evidence suggests that women want individualised information, particularly about their likeilihood of successful VBAC, to enable them to participate in the decision making process. This study aimed to identify characteristics that could inform a predictive model which would allow women to receive personalised and clinically specific information about their likelihood of achieving a successful VBAC in subsequent pregnancies.

**Methods:**

An observational study using anonymised clinical data extracted from a detailed, comprehensive socio-demographic and clinical dataset. All women who attempted a singleton term VBAC between 2000 and 2012 were included. Data were analysed using both logistic regression and Bayesian statistical techniques to identify clinical and demographic variables predictive of successful VBAC.

**Results:**

Variables significantly associated with VBAC were: ethnicity (*p* = 0.011), maternal obstetric complications (*p* < 0.001), previous vaginal birth (*p* = < 0.001), antepartum haemorrhage (*p* = 0.005), pre-pregnancy BMI (*p* < 0.001) and a previous second stage CS (*p* < 0.001).

**Conclusions:**

By using current literature, expert clinical opinion and having access to clinically detailed variables, this study has identified a new significant characteristic. Women who had a previous CS in the second stage of labour are more likely to have a successful VBAC. This predictor may have international significance for women and clinicians in shared VBAC decision-making. Further research is planned to validate this model on a larger national sample leading to further development of the nomogram tool developed in this study for use in clinical practice to assist women and clinicians in the decision-making process about mode of birth after CS.

## Background

Caesarean section (CS) rates are a public health concern globally, with as many as one in four women in the UK giving birth this way [1]. The global trend for CS delivery has a rising average annual increase of 4.4%, with rates in developed countries ranging from 40.5% in Latin America and the Carribean, to 19.2% in Asia [2]. CS rates also vary across the UK between different hospitals and geographical areas [3, 4].

The rise of overall CS in Scotland from 9% in 1976 to 32.4% in 2016 has provided no attributable decline in maternal or perinatal mortality [5, 6]. Furthermore, the rates of CS vary widely between 9.2 and 18.7% for elective procedures, and from 8.2 to 20% for emergency CS between different hospital sites in Scotland [5]. The increasing CS rate has inevitably resulted in a population of women with an obstetric history of CS and a consequent need for these women to make complex decisions about mode of birth after CS in subsequent pregnancies. Women who attempt a VBAC and have an emergency CS in labour encounter an increased risk of maternal and perinatal morbidity compared to those who chose an elective CS [7, 9].

Contemporary data indicates that almost 50% of CS are electively performed with a high proportion of these being a repeat procedure [1–3]. Vaginal birth after CS rates are declining, despite evidence that this is a safe option for most women and as many as three in four may be successful [3]. Policy and professional guidelines support seeking women’s preferences and decision-making being shared with the clinician about mode of birth after CS [4, 7, 8]. Evidence suggests that these variations are associated with professional attitudes and preferences when presenting women with information regarding mode of birth after a CS, women’s ability to have their individual decision making supported and the norms of each maternity care site [1, 3] . The increasing rate of CS may also have an impact on the experience of less senior obstetricians in counselling and the management in labour of women attempting vaginal birth after caesarean [10].

The quality of information received by women about options for their next birth after CS also varies. Many parts of the UK have established dedicated pathways of care for these women which aims to improve the access and consistency of information they receive [7]. Guidance is available to women and clinicians in the UK by the National Institute of Health and Care Excellence [7] and the Royal College of Obstetricians and Gynaecologists [4] regarding the risks and benefits of repeat elective CS and vaginal birth after CS for women who experienced an uncomplicated first CS in an otherwise normal pregnancy. The Montgomery ruling, which redefined the standard for informed consent and discolsure as to whether a reasonable person would attach significance to a risk, has resulted in an emphasis on counselling women about the potential complications of vaginal birth after caesarean [11]. A quality assessment of six available guidelines acknowledged the difficulties in providing full and unbiased information when worldwide evidence is still evolving [14]. Women’s decisions regarding mode of birth after caesarean has been perceived as difficult [13] and the information received has been described as a “fog” [14] of conflicting opinions and advice. Information that is tailored to women’s individual and evolving circumstances, taking into consideration their personal values and experiences, is sought by women and clinicians to inform their shared decision-making process [1, 12, 14–17].

Studies over the past decade attempt to define particular socio-demographic and clinical characteristics that may be used to predict women’s likelihood of achieving a vaginal birth after caesarean [1, 3, 19–21] and assist women’s decision-making regarding mode of birth after a CS [22, 23].

Models have been developed from these studies which identify predictive factors regarding the likelihood of a vaginal birth after caesarean. The majority of contemporary prediction models have been developed on non-European populations using large observational studies of heterogeneous women [4]. The need to develop a model based on data collected from the outcomes of women in their geographical context was recognised by Schoorel et al. [24] This led to the development of an internally validated model based on the outcomes of 515 Dutch women who attempted a vaginal birth after caesarean, providing a prediction model based on regression equations, which addressed the ethnic and obstetric policy context of a Western European population. Schoorel et al.’s [24] model has not, however, been validated on either a larger dataset or a UK population.

Two methods of analysis are commonly used in order to provide predictive scores in healthcare. These are regression analysis and Bayesian modelling. Regression analysis is used to predict an unknown value of a dependent variable from the known values of two or more independent variables [25, 26]. Alternatively, Bayesian networks provide an intelligent system that fuses information from different sources and is able to represent and manipulate uncertainties from prior data, assimilating more data as it becomes available, and assign a posterior distribution of probability to a hypothesis [25].

Whilst both methods have been found to be useful in providing a prediction score or a probability scoring in clinical outcomes, [26–29] no studies have been found which compare regression analysis and Bayesian modelling as methods of analysis to find which is the most reliable in predicting a successful vaginal birth.

The aim of the study was to identify predictive characteristics that could inform a predictive model which would allow women to receive personalised and clinically specific information about their likelihood of achieving a vaginal birth in subsequent pregnancies.

## Methods

Aberdeen Maternity and Neonatal databank (AMND), holds data for all births in a tertiary Scottish referral centre from 1949 to the present day.

The data held in the AMND on individual women allowed variables from pregnancies prior to the attempted VBAC (where mode of birth was CS) and the pregnancy when VBAC was attempted to be explored. The detailed data available from AMND allowed a wide variety of potentially influential variables to be analysed. These included socio-demographic (for example age, age at both deliveries, inter-delivery interval, ethnicity and social class) clinical (for example reason for previous CS, modes of birth in any other previous pregnancies, antenatal complications, pre-exisiting medical conditions, length of 1st and 2nd stage of labour, gestation, sex and birthweight of baby, spontaneous or induced onset of labour, prolonged rupture of membranes) for both pregnancies. In all 120 variables for each woman (60 for each pregnancy) were included which provided the data on which the prediction scores and Bayesian probability models described in this paper were developed and internally validated. The most reliable predictive model for use clinically to support women and clinicians in the decision-making process regarding mode of birth after CS throughout pregnancy, whether by Bayesian or frequentist methods of analysis has yet to be determined.

Following appropriate ethical permissions and a successful application to access data from the AMND, data were extracted from consecutive records held within the AMND from 1.1.2000 to 31.12.2012. All women with a history of one or more previous lower segment CSs, with any number of prior vaginal births, and a subsequent attempt to achieve a vaginal birth after caesarean in the next pregnancy after CS were included. To ensure the model was theoretically informed, potential variables were preselected from published prediction models. Expert clinical opinion regarding the data variables to be collected was also sought from senior obstetric and midwifery clinicians.

The data were checked for validity for each variable using range and internal consistency checks through cross tabulation. The outcomes were successful vaginal birth after caesarean and emergency repeat caesarean. Full data sets were available for the majority of the potential predictors, but 3% of women had no Body Mass Index (BMI) recorded. After imputation, as used by Schoorel et al. [24] multiple sets of plausible values were allocated to this variable only, and all women were available for multivariate modelling.

### Regression equation analysis

The summary measures were mean (standard deviation) for normally distributed data, median (inter-quartile range) for non-normally distributed or skewed data and count (percentile) for categorical data for each of the outcome groups. Bivariate and univariate analyses were used to find associations between various maternal and fetal factors and vaginal birth after caesarean. Appropriate chi-square testing was applied to find any association between obstetric history and perinatal factors, and the outcomes. Independent t-test or Mann-Whitney U test, wherever appropriate [26], were used to compare pre-pregnancy BMI between successful vaginal birth after caesarean and emergency CS. Those plausible predictors which were significant at 20% level (*p* < 0.2) in the bivariate analysis and proven or suspected to be of clinical importance were selected for further consideration in the model development. A bootstrap method with backward elimination was used to examine stability of the predictors in the model. The variables with bootstrap inclusion fraction of more than 60% were included in further multivariate modelling.

### Bayesian analysis

Univariate Bayesian logistic regression modes were fitted for variables that had previously been identified as potentially useful through bootstrapping within the frequentist framework described above. A burn in phase of 10,000 iterations were selected to allow the model to converge. The estimates of the regression coefficients were based on a further 10,000 iterations. Convergence of the Bayesian model estimates was assessed through Gelman Rubin [30] statistic plots obtained from two sets of initial values.

From univariate analysis, only those variables with 80% credible intervals excluding zero were selected for inclusion in the multivariate analysis. This was equivalent to using a frequentist analysis with a *p* < 0.2 criterion. The 80% credible intervals presented were based on only one set of initial values.

For each regression coefficient (log odds ratio) the median of the 10,000 estimates and the 95% credible interval were calculated. The exponential was taken of these summary statistics so that those equivalents to the odds ratios could be compared to those from the frequentist analyses.

**Fig. 1:**
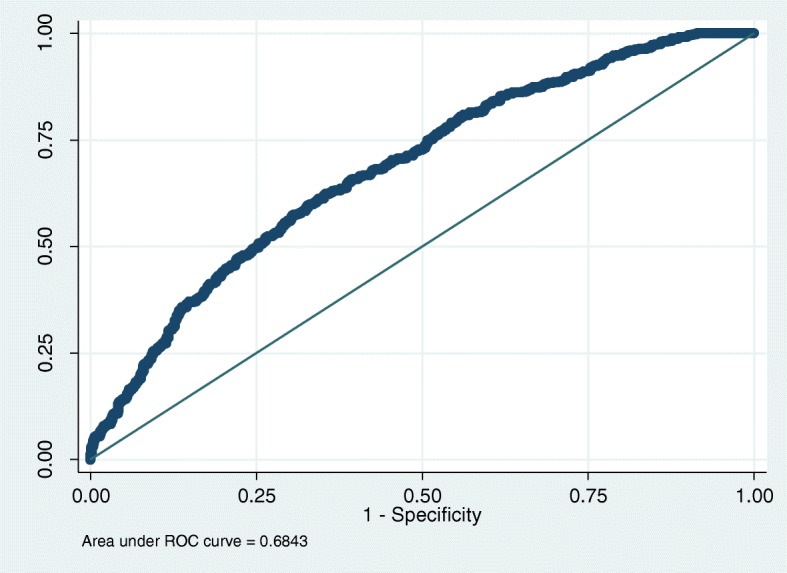
Area under the ROC curve for probability of vaginal birth after caesarean

The variables found to be predictive of vaginal birth after caesarean in both forms of analysis were further tested by determining their discriminative ability. Discrimination refers to the ability to distinguish women with a high to a low probability of achieving a vaginal birth after caesarean. The index of discrimination is commonly expressed by measure of concordance, the c-statistics which are identical to the area under the receiver operating characteristic (ROC) curve and varies between 0.5 and 1 [31]. If a pair of women are randomly drawn from the study data and the observed outcomes are different, a woman who achieved a successful vaginal birth after caesarean will have a higher predicted probability compared to another woman with unsuccessful vaginal birth after caesarean. The predictions in this model were found to have concordance with the observed outcome (Fig. 1).

Statistical analyses were performed using the STATA data analysis and statistical software version 14 MP.

## Results

A total of 1661 women who attempted a vaginal birth in the next pregnancy after a CS, were identified as eligible and were included in this study. Most women (906, 54.5%) had a repeat CS in labour and only 755 (45.5%) women had a successful vaginal birth. The number of socio- demographic and clinical characteristics (categorical and continuous variables) in this cohort likely (at *p* value > 0.2) to be significant in predicting a vaginal birth after caesarean, are shown in Tables 1 and 2.

**Table 1:** Demographic Characteristics found to have an association with VBAC

Mode of Birth	Vaginal Birth		Caesarean Section		*p*-value
	*n* = 775		*n* = 906		
Characteristics	No	%	No	%	
Age in years					
Under 20	8	80	2	20	0.153
20–24	58	50.9	56	49.1	
25–29	166	44.4	208	55.6	
30–35	300	45.6	358	54.4	
35+	223	44.2	282	55.8	
Missing	241	46.0	269	52.7	
Age Difference between CS and attempted VBAC Birth					
< =5 years	694	46.1	811	53.9	0.112
> 5 years	134	35.8	240	64.2	
Pregnancy Number					
2	472	47.1	530	52.9	< 0.001
3	176	45.7	209	54.3	
4+	107	39.1	167	60.9	
Ethnicity					
White Caucasian	730	46.0	856	54	0.059
Black	9	37.5	15	62.5	
Asian	12	3.0	32	72.7	
Other	4	3.0	3	42.9

**Table 2:** Clinical Characteristics foound to have an association with VBAC

Mode of Birth	Vaginal Birth			Caesarean Section			*p* -value
	*n* = 775			*n* = 906			
Characteristics	No		%	No		%	
Maternal Complications							
No	621		48.3	666		51.7	< 0.001
Yes	134		35.8	240		64.2	
Mode of births prior to CS							
No Previous births	655		46.6	751		53.3	< 0.001
Previous Vaginal Birth	91		58.7	64		41.3	
Previous CS	9		9.0	91		91.0	
Previous CS Reason							
No Reason Recorded	16		44.4	20		55.6	< 0.001
Non-progressive Labour	319		41.6	448		58.4	
Fetal Compromise	155		45.3	187		54.7	
Other Complications	37		29.6	88		70.4	
Breech Presentation	228		58.3	163		41.7	
Antepartum Haemorrhage							
No	673		46.8	765		51.3	0.005
Yes	82		36.8	141		63.2	
Reached 2nd Stage of Labour							
No	617		43.3	809		56.7	<0.001
Yes	138		58.7	97		41.3	
BMI at Booking (categorical)	No	Mean	SD	No	Mean	SD	
BMI at Booking	775	25.8	5.02	906	27.23	5.78	<0.001

Factors associated with successful vaginal birth after caesarean were found to be a previous CS performed before the onset of labour, or ever having had a vaginal birth prior to CS, a normal or low booking BMI, white ethnicity, the previous CS for breech presentation, reaching the second stage of labour, no maternal complications and no history of antepartum haemorrhage. There was no evidence of multicollinearity or interaction between variables. The prediction ability of the model was assessed through discrimination (AUROC = 0.68) and calibration with *p* = 0.57 for Hosmer-Lemeshow [32] test for goodness of fit.

**Fig. 2:**
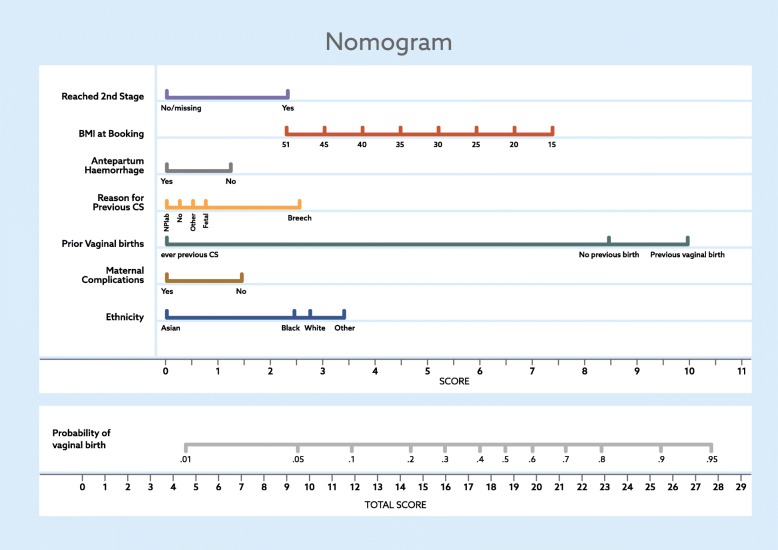
Grampian women’s probability of vaginal birth after caesarean nomogram

The nomogram (Fig. 2) was developed using both the response of predictors and an allocated corresponding score (bottom axis) for ease of use in clinical practice. For example, a woman’s BMI of 51 contributes approximately 2 points in the score; this is determined by comparing the location of the BMI to the points in the score and drawing vertical line between the two axes. The point values from the score of all predictors are determined in a similar manner and are summarised to arrive at a ‘Total Score’. This value is plotted on the ‘Total Score’. A vertical line drawn from the total score straight up to the probability scale will indicate the woman’s probability of a successful vaginal birth after caesarean.

## Discussion

The socio-demographic and clinical characteristics described in the literature to be positively or negatively associated with the likelihood of successful vaginal birth after caesarean were very similar in both the Bayesian and the logistic regression analysis of this study. Vaginal birth prior to CS, breech presentation as a reason for the previous CS and a normal BMI were all positively associated with achieving a vaginal birth after caesarean in a subsequent pregnancy. Previous CS in the second stage of labour was uniquely identified in this study as a new variable of clinical significance which was also strongly associated with a successful vaginal birth after caesarean. Being of Asian ethnic group (including China and the Indian sub continent), experiencing maternal complications, ever having had a CS in a previous pregnancy particularly for non-progressive labour, a higher BMI in subsequent pregnancies and experiencing an ante-partum haemorrhage were all associated with less likelihood of achieving a vaginal birth after caesarean in subsequent pregnancies.

Whilst most of the strongest predictors of vaginal birth in this study have already been described in the literature, reaching the second stage of labour had not previously been identified. Non-progressive labour as a reason for the previous CS is used as a negative and potentially recurrent predictor of successful vaginal birth after CS in guidance and models in current use [12, 20, 21, 24, 25]. Reaching the second stage may be seen as a proxy measure for a progressive labour, but the level of detail in our data has allowed a clear indication that once this has been achieved, women have a statistically greater chance of successful vaginal birth after CS. Previous studies have attempted to determine the maximum degree of cervical dilation achieved to predict the likelihood of successful vaginal birth after CS in the future [35]. Hoskins and Gomez [37] reported a low success rate (13%) when the previous CS was performed at a cervical dilatation of 9cms but Stronge et al. [38] suggested that increased cervical dilatation on admission was indicative of significantly increased likelihood of vaginal birth after caesarean which indicates an alignment with the findings of this study.

Contemporary studies [20, 24, 36, 39] using retrospective and prospective cohorts have all found that the greatest predictor for successful vaginal birth after CS was a previous vaginal birth, whether before or after CS. The findings of this study indicated that women who had no previous vaginal births before their CS, as well as those who had experienced a previous vaginal birth were more strongly predictive of a vaginal birth after CS than any other factor. It would seem that the definitive factor may be the absence of a history of non-progressive labour and may reflect a conservative approach to informing women’s decision making about the likelihood of achieving a vaginal birth after a previous CS and avoiding an emergency CS for those who previously experienced a non-progressive labour [4, 19].

The BMI of the women at booking in the pregnancy that vaginal birth after CS was attempted was found to have a significant influence on the likelihood of successful vaginal birth after CS in this study. This finding concurs with Callegari et al’s [18] conclusions that women can improve their chance of successful vaginal birth after CS by achieving or maintaining a healthy BMI between pregnancies.

Few women in this study were of non-British ethnicity, reflecting the demographics of the population. This variable was found to be predictive of successful vaginal birth after CS and further supports published prediction models [21, 24, 39] which suggest a success rate of over 70% for white European women.

The lower vaginal birth after caesarean success rate found in this study in comparison with recent studies [3] of 63.4%, in England, 65% in America [19] and 72% in the Netherlands [24], could be a result not only of our inclusion of women with more than one previous CS, but also of clinicians adopting a more cautious approach to the management of women who attempt a vaginal birth after CS, for example through greater willingness to resort to emergency caesarean if progress in labour is slow. Most clinicians also now avoid using augmentation of labour for women attempting vaginal birth after CS [4, 33, 34, 40], as there is growing evidence that this can increase the risk of uterine rupture.

The systematic review by Eden et al. [34] suggested that prediction of failure of achieving an outcome is less accurate in published models and calls for a model that incorporates known obstetric factors but can be adapted for evolving changes in women’s clinical circumstances which the positive associations in this model aims to provide.

The strengths of this study are the comprehensive and consistency of the of the data used, the unique use of two different statistical methods of analysis on the same dataset, and the inclusion of all women who require information to inform their choice of mode of birth after CS. The sample size for this study (*n* = 1661) was three times greater than that previously used in studies specifically exloring prediction of successful VBAC in a European population. The large population database used as the data source contained detailed data on all women’s pregnancies in the geographical area over the past 60 years. The data is entered into the Aberdeen Maternity and Neonatal Database by an experienced team who code data directly from medical records. The quality of the data entry is monitored to maintain a consistently high-quality dataset. Only 3% of data were missing and none of the variables selected were excluded due to data issues which reduced the likely sources of bias in this study.

Information on variables identified by previous studies and unique clinical characteristics highlighted by expert clinicians which may be clinically relevant were available in the database and included in the analysis. Over 60 variables were initially included in our analysis and this is the first study to use the Aberdeen Maternal and Neonatal Database to explore the determinants of successful vaginal birth after CS to provide information for all women and clinicians aimed at assisting decision making about mode of birth after CS.

This is the first model built on Scottish data. It provides a more comprehensive exploration of predictive characteristics compared to other UK models [1, 3], in that it is based on the population of all women who make the decision about mode of birth after CS, rather than only those who have a history of only one previous CS. The theoretical model has not yet been tested on a different data set. This will be performed on a national dataset in the next phase of this study. Both the multiple regression and Bayesian methods of analysis arrived at the same factors of relevance for women’s predicted chance of achieving a vaginal birth after CS. This homogeneity would help strengthen the case for the validity of the analysis in that we found an overall consistency in these factors whichever analytical approach, whether based on frequentist or probability theoretical backgrounds, was taken.

The retrospective design of this study is associated with limitations of the use of data routinely collected, rather than data tailored specifically to the study objectives. In the absence of large, randomised clinical trials which are considered to be unfeasible [35], this study provides an important contribution to the evidence about the likelihood of successful vaginal birth after CS for all women who are faced with the decision of opting for elective repeat CS or attempting a vaginal birth after CS. The characteristics identified by this research were derived from data collected from women in a wide range of clinical circumstances which may have wide reaching applications in the global context of developed countries.

Previous studies have made recommendations for a defined population of women with one previous CS in an uncomplicated pregnancy. This research has included data from the variety of women that are more representive of those using clinical services who are seeking information particularly on their likelihood of successful vaginal birth after CS. The nomogram informed by the characteristics identified in this study has taken into consideration the wide range of individual medical and obstetric histories and subsequent clinical complications of pregnancy to help inform the complex decisions that are made by women and clinicians in practice.

## Conclusion

This study has revealed a clinically significant characteristic which adds new information to the evidence on determinants of successful vaginal birth after CS. The use of clinically detailed data from a large database and the input of expert clinicians as well as the literature in the selection of the characteristics has facilitated a comprehensive exploration of the data available. This ability to use potentially previously unexplored characteristics, coupled with the novel approach of using two different but appropriate methods of analysis, has revealed new clinically significant information which would have worldwide relevance. Whilst validating clinical characteristics previously identified in the worldwide literature, undergoing a previous CS in the second stage of labour can also be seen as a predictive factor positively associated with successful vaginal birth after CS.

Further research is planned to validate this model on a larger national sample leading to further development of the nomogram tool for use in clinical practice to assist women and clinicians in the decision-making process about mode of birth after CS.

## Abbreviations

AMND: Aberdeen Maternal and Neonatal Database
BMI: Body Mass Index
CS: Caesarean Section
NHS: National Health Service
VBAC: Vaginal birth after Caesarean
